# In vivo neuroinflammation and cerebral small vessel disease in mild cognitive impairment and Alzheimer’s disease

**DOI:** 10.1136/jnnp-2020-323894

**Published:** 2020-09-11

**Authors:** Audrey Low, Elijah Mak, Maura Malpetti, Luca Passamonti, Nicolas Nicastro, James D Stefaniak, George Savulich, Leonidas Chouliaras, Li Su, James B Rowe, Hugh S Markus, John T O'Brien

**Affiliations:** 1 Department of Psychiatry, University of Cambridge, Cambridge, UK; 2 Department of Clinical Neurosciences, University of Cambridge, Cambridge, UK; 3 Department of Clinical Neurosciences, Division of Neurology, Geneva University Hospitals, Geneva, Switzerland; 4 Division of Neuroscience and Experimental Psychology, The University of Manchester, Manchester, UK; 5 MRC Cognition and Brain Sciences Unit, University of Cambridge, Cambridge, UK

## Abstract

**Introduction:**

Associations between cerebral small vessel disease (SVD) and inflammation have been largely examined using peripheral blood markers of inflammation, with few studies measuring inflammation within the brain. We investigated the cross-sectional relationship between SVD and in vivo neuroinflammation using [^11^C]PK11195 positron emission tomography (PET) imaging.

**Methods:**

Forty-two participants were recruited (according to NIA-AA guidelines, 14 healthy controls, 14 mild Alzheimer’s disease, 14 amyloid-positive mild cognitive impairment). Neuroinflammation was assessed using [^11^C]PK11195 PET imaging, a marker of microglial activation. To quantify SVD, we assessed white matter hyperintensities (WMH), enlarged perivascular spaces, cerebral microbleeds and lacunes. Composite scores were calculated for global SVD burden, and SVD subtypes of hypertensive arteriopathy and cerebral amyloid angiopathy (CAA). General linear models examined associations between SVD and [^11^C]PK11195, adjusting for sex, age, education, cognition, scan interval, and corrected for multiple comparisons via false discovery rate (FDR). Dominance analysis directly compared the relative importance of hypertensive arteriopathy and CAA scores as predictors of [^11^C]PK11195.

**Results:**

Global [^11^C]PK11195 binding was associated with SVD markers, particularly in regions typical of hypertensive arteriopathy: deep microbleeds (β=0.63, F(1,35)=35.24, p<0.001), deep WMH (β=0.59, t=4.91, p<0.001). In dominance analysis, hypertensive arteriopathy score outperformed CAA in predicting [^11^C]PK11195 binding globally and in 28 out of 37 regions of interest, especially the medial temporal lobe (β=0.66–0.76, t=3.90–5.58, FDR-corrected p (p_FDR_)=<0.001–0.002) and orbitofrontal cortex (β=0.51–0.57, t=3.53–4.30, p_FDR_=0.001–0.004).

**Conclusion:**

Microglial activation is associated with SVD, particularly with the hypertensive arteriopathy subtype of SVD. Although further research is needed to determine causality, our study suggests that targeting neuroinflammation might represent a novel therapeutic strategy for SVD.

## Introduction

Cerebral small vessel disease (SVD) and neuroinflammation are increasingly recognised as key contributors to Alzheimer’s disease (AD) and other neurodegenerative disorders.^1–3^ Both SVD and neuroinflammation have been shown to promote neurodegeneration and worsen its clinical consequences, although their pathophysiological mechanisms remain unclear.^3–7^

Neuroinflammation plays a central role in AD and is increasingly recognised as an early event in its pathogenesis rather than a response to late stage disease.^2 3^ Under normal circumstances, inflammation acts as a natural physiological defence against infections and injury, thereby playing a neuroprotective role. Ordinarily, the immune response undergoes a process of *activation*, whereby microglia are activated to combat infection, followed by *resolution*, with microglia returning to ‘resting’ state. However, this process can become dysfunctional, such that resolution is not achieved. This results in a chronic state of inflammation marked by sustained activation of microglia, and excessive, dysregulated cytokine production, which can have deleterious effects on brain tissue, endothelial function and the cerebrovascular network.^8^

Similarly, SVD has also been associated with increased AD risk^1 9^ and can be detected on MRI in the form of white matter hyperintensities (WMH), lacunes, cerebral microbleeds (CMB) and enlarged perivascular spaces (EPVS).^10 11^ Aetiologically, these SVD-related brain changes are thought to be ischaemic in nature, resulting from arteriolar narrowing or occlusion. However, these arteriolar changes could be a late-stage phenomenon and may not explain early pathology potentially arising from other pathological processes.^7^ Notably, neuroinflammation has been recently proposed as another candidate mechanism that can promote or accelerate SVD.^12^

Despite the well-established role of both inflammation and SVD in dementia, investigations on the relationship between the two mechanisms have been largely limited to the use of peripheral blood markers of inflammation (eg, C-reactive protein, fibrinogen),^12^ although some neuropathological evidence also exist.^13^ Unfortunately, peripheral markers may not be reflective of inflammation within the central nervous system (CNS) and lack spatial information on the distribution of neuroinflammation within the brain.^5^ As such, in vivo measures of neuroinflammation and topographical analysis are required to further our understanding of the pathological processes underlying AD and other neurodegenerative disorders.

In a recent systematic review, we found that serum-based measures of inflammation were differentially related to two distinct forms of SVD.^12^ First, markers of *vascular* inflammation and endothelial dysfunction were preferentially associated with SVD in regions typically affected by hypertensive arteriopathy (eg, basal ganglia). Conversely, *systemic* inflammatory markers were positively related to SVD in regions that are characteristically compromised in cerebral amyloid angiopathy (CAA) (eg, lobar microbleeds). These distinct forms of SVD are represented by Pantoni’s aetiological classification of type 1 and type 2 SVD.^14^ Hypertensive arteriopathy is characterised by vascular injury in regions supplied by deep perforating arteries and associated with deep lacunes and microbleeds, and EPVS in the basal ganglia.^15–18^ By contrast, CAA involves vascular alterations in lobar/cortical regions; these regions are supplied by cortical and leptomeningeal vessels, which are commonly affected by CAA through the deposition of Aβ within these vessel walls.^11 14 15^

In this study, we examined the association between SVD and in vivo CNS measures of neuroinflammation using [^11^C]PK11195 positron emission tomography (PET), analysing both global severity and regional distribution of these biomarkers. [^11^C]PK11195 is a ligand of the 18 kDa translocator protein (TSPO, or peripheral benzodiazepine receptor) and a well-established PET marker of microglial activation.^5^ Our overarching hypothesis was that neuroinflammation would be positively associated with SVD and that the degree of inflammation would relate to SVD severity. In acknowledging the different aetiologies between SVD subtypes, we further hypothesised that neuroinflammation would be more strongly related to hypertensive arteriopathy rather than CAA, given the stronger vascular underpinnings of the former (eg, hypertension, diabetes, endothelial dysfunction), compared with the latter, which is thought to be driven by Aβ deposition.^14^

## Methods

Forty-two participants (14 healthy controls, 14 amyloid-positive mild cognitive impairment (MCI), 14 early AD) above age 50 were recruited within the Neuroimaging of Inflammation in Memory and Other Disorders protocol (see details in online supplemental materials).^19^ MCI was defined as Mini-Mental State Examination (MMSE) >24, but with memory impairments beyond what is expected for age and education which did not meet the criteria for probable AD and was not explained by another diagnosis. Due to the heterogeneity in the subtypes and causes of MCI, only amyloid-positive MCI patients were included in this study to represent a preclinical stage of AD—this was defined by an average cortical ^11^C Pittsburgh Compound-B standardised uptake value ratio above 1.5. Probable AD was defined according to the National Institute on Aging-Alzheimer’s Association (NIA-AA) guidelines.^20^ AD and amyloid-positive MCI participants were combined into a single group on the basis that these groups represent a continuum of the same clinical spectrum. Healthy controls were required to have MMSE >26, be devoid of cognitive symptoms or unstable/significant medical illness. Global cognition was assessed using the MMSE and Addenbrooke’s Cognitive Examination-Revised (ACE-R), and episodic memory was examined using the Rey Auditory Verbal Learning test.

10.1136/jnnp-2020-323894.supp1 Supplementary data

### MRI acquisition

All participants underwent 3T MRI scanning (acquisition parameters are detailed in online supplemental materials). T1-weighted images were non-rigidly registered to the ICBM2009a template brain using ANTS (www.picsl.upenn.edu/ANTS/) and inverse transform was applied to a modified Hammers atlas (resliced from MNI152 to ICBM2009a space) to bring regions of interest (ROI) to subject space, to which PET data described later were co-registered.^4 6 21^

### PET imaging protocols

To measure the degree of neuroinflammation in the brain, all participants underwent [^11^C]PK11195 PET imaging (see acquisition and processing details in online supplemental materials. Binding in each ROI was quantified using non-displaceable binding potential (BP_ND_) determined with a simplified reference tissue model incorporating vascular binding correction and reference region time activity curve estimation from supervised cluster analysis using four kinetic classes.^22^ Regional BP_ND_ was corrected for cerebrospinal fluid contamination through division of the ROI time activity curve with the mean ROI fraction of grey matter and white matter (WM), using Statistical Parametric Mapping V.8 (SPM8; www.fil.ion.ucl.ac.uk/spm/) probability maps smoothed to match the PET spatial resolution. Volume-weighted average [^11^C]PK11195 binding was computed for each individual.

### Quantification of SVD

#### Semi-quantitative measurements

**Figure 1:**
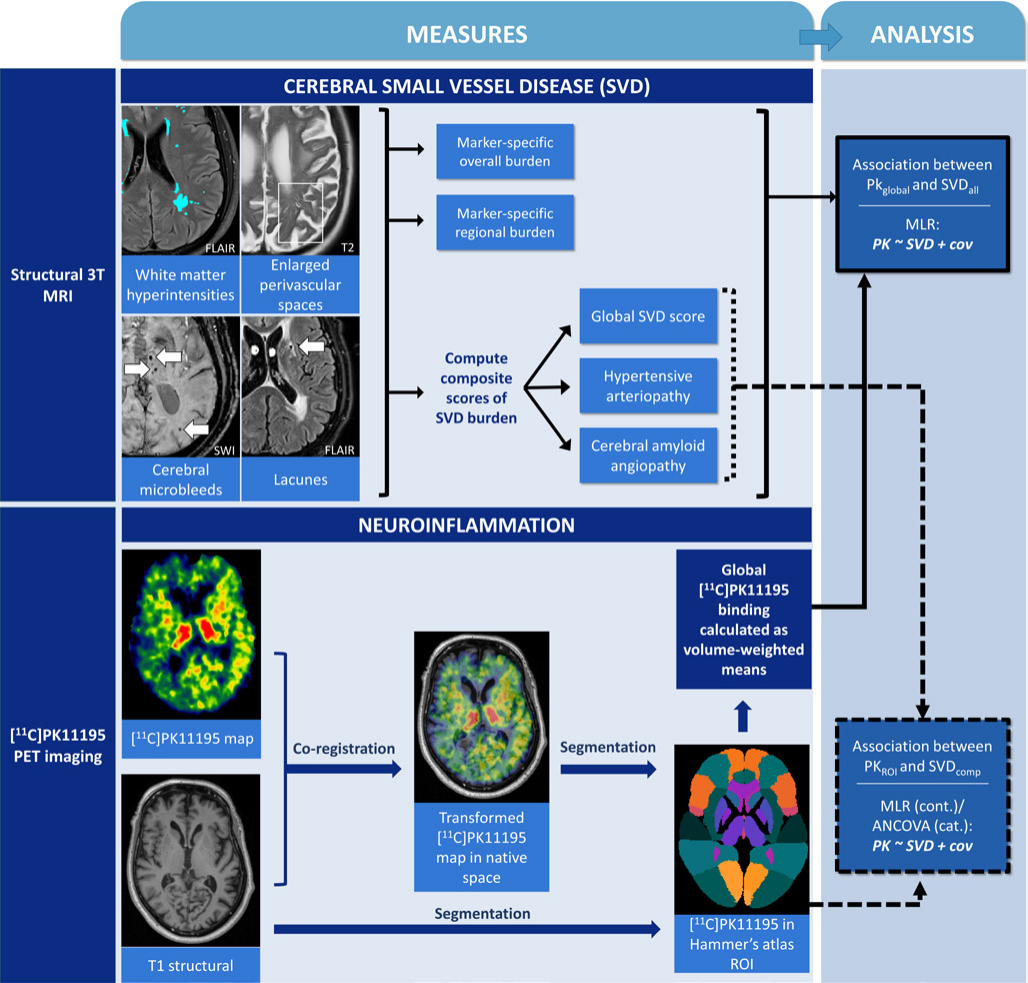
Schematic representation of multimodal imaging data, image processing pipeline, quantification of pathological burden and statistical approach to test associations between pathologies. ACE-R, Addenbrooke’s Cognitive Examination-Revised; cat., categorical measures, for example, presence/absence of microbleeds and lacunes; cov, covariates (sex, age, education, ACE-R, and scan interval); cont., continuous SVD measures; MLR, multiple linear regression; PK_global_, global [^11^C]PK11195 binding; PK_ROI_, [^11^C]PK11195 binding in each Hammer’s atlas region of interest; SVD_all_, each measure of SVD, including global and regional data of each SVD marker, and composite SVD scores; SVD_comp_, composite SVD scores; SVD, small vessel disease.

SVD markers were manually rated on structural MRI scans (figure 1). WMH were visually rated on fluid-attenuated inversion recovery (FLAIR) MRI according to the Fazekas scale (online supplemental materials).^23^ EPVS were rated on T2-weighted MRI using a validated rating scale^24^; basal ganglia and centrum semiovale EPVS scores ranged from 0 to 4 according to EPVS count: 0 (none), 1 (1–10), 2 (11–20), 3 (21–40) and 4 (>40), while midbrain EPVS were rated dichotomously (present/absent). CMB were assessed on susceptibility-weighted imaging following the Microbleed Anatomical Rating Scale.^25^ Lacunes were identified using T1-weighted, T2-weighted and FLAIR, following the STandards for ReportIng Vascular changes on nEuroimaging guidelines.^11^ Lacunes and microbleeds were classified according to location as deep (eg, basal ganglia, thalamus) or lobar (eg, centrum semiovale) lesions. Lacunes and CMB data were dichotomised separately as ‘present’ (at least one lesion) or ‘absent’ (no lesions).

#### Quantitative SVD measurements

WMH volumes were obtained using an automated script on the SPM8 suite; details on the procedures involved have been described previously^26^ and are detailed under online supplemental materials. To account for individual differences in head size, WMH volumes were normalised by total intracranial volume.

#### Global SVD burden scores

A total SVD burden score was computed using a point system based on the presence or absence of each of the four SVD markers, according to cut-offs defined by Staals and colleagues (table 1).^27^ To examine [^11^C]PK11195 in relation to different subtypes of SVD, CAA and hypertensive arteriopathy scores (range 0–4 points) were formulated according to distinctions made in the literature,^15–18 28^ assigning one point per criterion met (table 1; online supplemental materials).

**Table 1:** Scoring of global cerebral SVD burden, hypertensive arteriopathy and cerebral amyloid angiopathy scores

		Global SVD (Staals *et al*)^27^	Hypertensive arteriopathy	Cerebral amyloid angiopathy
One point per criterion met	WMH	Periventricular WMH=3 and/or deep WMH=2 or 3	Deep WMH=2 or 3	Periventricular WMH=3 and/or deep WMH=2 or 3
	EPVS	EPVS rating in basal ganglia ≥2	EPVS rating in basal ganglia ≥2	EPVS rating in centrum semiovale ≥2
	CMB	CMB present	Deep CMB present	Lobar CMB present
	Lacunes	Lacunes present	Deep lacunes present	Lobar lacunes present

CMB, cerebral microbleeds; EPVS, enlarged perivascular spaces; SVD, small vessel disease; WMH, white matter hyperintensities.

### Lesion probability mapping

To examine the spatial patterns of WMH associated with global [^11^C]PK11195 binding, we used a non-parametric permutation-based method implemented in FSL-PALM, adjusting for sex, age, education, ACE-R and scan interval between PET and MRI and modelling the intercept (10 000 permutations); technical details were previously described.^29^ Statistically significant clusters were defined using threshold-free cluster enhancement, followed by family-wise error rate (FWER) correction (p_FWER_ <0.05), thus avoiding the need for an arbitrary cluster-forming threshold.

### Statistical analysis

Statistical analysis was performed using R (https://www.r-project.org/). Standard statistical techniques were used for descriptive analyses (table 1) (see details in online supplemental materials). Adjusted analysis for continuous variables were conducted using general linear modelling. Assumptions of all linear regression models were first tested for skewness, heteroscedasticity and kurtosis and were validated to have met each linear model assumption. To reduce collinearity, all independent variables in linear models were mean-centred. All regressions were adjusted for the same set of covariates: sex, age, years of education, ACE-R score (to adjust for disease severity) and duration between MRI and PET scans. Given its relevance to both neuroinflammation and SVD, history of hypertension was included as a covariate in a separate step to distil its effects. To account for other vascular risk factors, a composite vascular risk score was computed (range 0–4 points), whereby one point was assigned for the presence of each of the following: hypertension, hyperlipidaemia, diabetes mellitus and current smoker. This vascular risk score was also entered as an additional covariate in a separate regression model. To examine associations between global [^11^C]PK11195 binding and SVD, general linear models were constructed with [^11^C]PK11195 entered as the predictor alongside the aforementioned covariates and SVD measures (WMH, EPVS, global SVD, hypertensive arteriopathy and CAA scores) as the outcome variable in separate models, while analysis of covariance was performed to analyse [^11^C]PK11195 in relation to the presence/absence of CMB and lacunes. False discovery rate (FDR) correction was applied for multiple comparisons. Global SVD, hypertensive arteriopathy and CAA scores were analysed in relation to regional binding of [^11^C]PK11195 across all bilateral Hammer’s atlas ROIs, excluding the ventricles, cerebellum and brainstem (37 ROIs) using general linear modelling controlling for all covariates and FDR-corrected. To directly compare hypertensive arteriopathy and CAA on the strength of their relationship with [^11^C]PK11195, dominance analysis was conducted. Dominance analysis improves on earlier methods of assessing relative contribution (eg, standardised coefficients, squared beta weights, squared zero-order correlations) due to its ability to account for correlations between predictors in multivariate analysis,^30^ an important feature given the potential overlap between CAA and hypertensive arteriopathy.

## Results

Participant characteristics are presented in table 2. The overall sample had a mean age of 71.6 (SD 8.0) years and an average of 13.5 (SD 3.0) years of education. Healthy controls and AD/MCI groups were comparable on sex, age and education. As expected, AD/MCI participants performed significantly worse than healthy participants on measures of cognition (table 2). AD/MCI participants had greater WMH volumes than healthy participants, although groups were comparable on global [^11^C]PK11195 binding and other SVD measures. Sixteen participants (43%) scored ≥2 on the CAA scale, while 8 (19%) scored ≥2 on the hypertensive arteriopathy scale. The two SVD scales were positively correlated (r=0.549, p<0.001).

**Table 2:** Participant characteristics

		Healthy controls	AD/MCI	P value
N		**14**	**28**	
Demographics				
Sex†	% Female	42.9%	42.9%	1.000
Age (years)‡	Mean (SD)	70.4 (6.4)	72.2 (8.7)	0.480
Education (years)§	Mean (SD)	14.4 (2.8)	13.1 (3.1)	0.147
Hypertension†	% Present	21.4%	25.0%	0.798
Hyperlipidaemia†	% Present	14.3%	33.3%	0.192
Diabetes mellitus†	% Present	7.1%	7.1%	1.000
History of smoking†	% Present	50.0%	17.9%	0.030*
Current smoker†	% Present	0.0%	7.1%	0.306
Cognition				
MMSE§	Mean (SD)	28.9 (1.1)	25.3 (2.5)	<0.001***
ACE-R‡	Mean (SD)	93.5 (4.4)	77.8 (8.9)	<0.001***
RAVLT‡	Mean (SD)	44.6 (9.1)	25.0 (8.7)	<0.001***
Imaging features				
WMH volume§¶	Mean (SD)	0.28 (0.26)	0.73 (0.58)	0.017*
Whole brain(^11^C)PK11195‡	Mean (SD)	0.04 (0.03)	0.06 (0.05)	0.197
EPVS (basal ganglia)††	Mean (SD)	1.43 (0.65)	1.54 (0.74)	0.678
CMB†	% Present	42.9%	32.1%	0.495
Lacunes†	*% Present*	42.9%	46.4%	0.826
Global SVD score (0–4)††	Mean (SD)	1.43 (1.22)	1.79 (1.34)	0.443
CAA score (0–4)††	Mean (SD)	1.00 (1.04)	1.57 (1.40)	0.235
HA score (0–4)††	Mean (SD)	0.57 (0.85)	0.96 (1.07)	0.254

*p<0.05; ***p<0.001.

†χ2 test of independence.

‡T-test.

§Mann-Whitney U test.

¶Normalised volume adjusted for TIV: (volume in mL/TIV) × 100%.

††Kruskal-Wallis test.

ACE-R, Addenbrooke’s Cognitive Examination-Revised; AD, Alzheimer’s disease; CAA, cerebral amyloid angiopathy; CMB, cerebral microbleeds; EPVS, enlarged perivascular spaces; HA, hypertensive arteriopathy; MCI, mild cognitive impairment; MMSE, Mini-Mental State Examination (amyloid-positive); RAVLT, Rey Auditory Verbal Learning Test; SVD, small vessel disease; TIV, total intracranial volume; WMH, white matter hyperintensities.

### Association between whole-brain [^11^C]PK11195 and SVD markers

**Figure 2:**
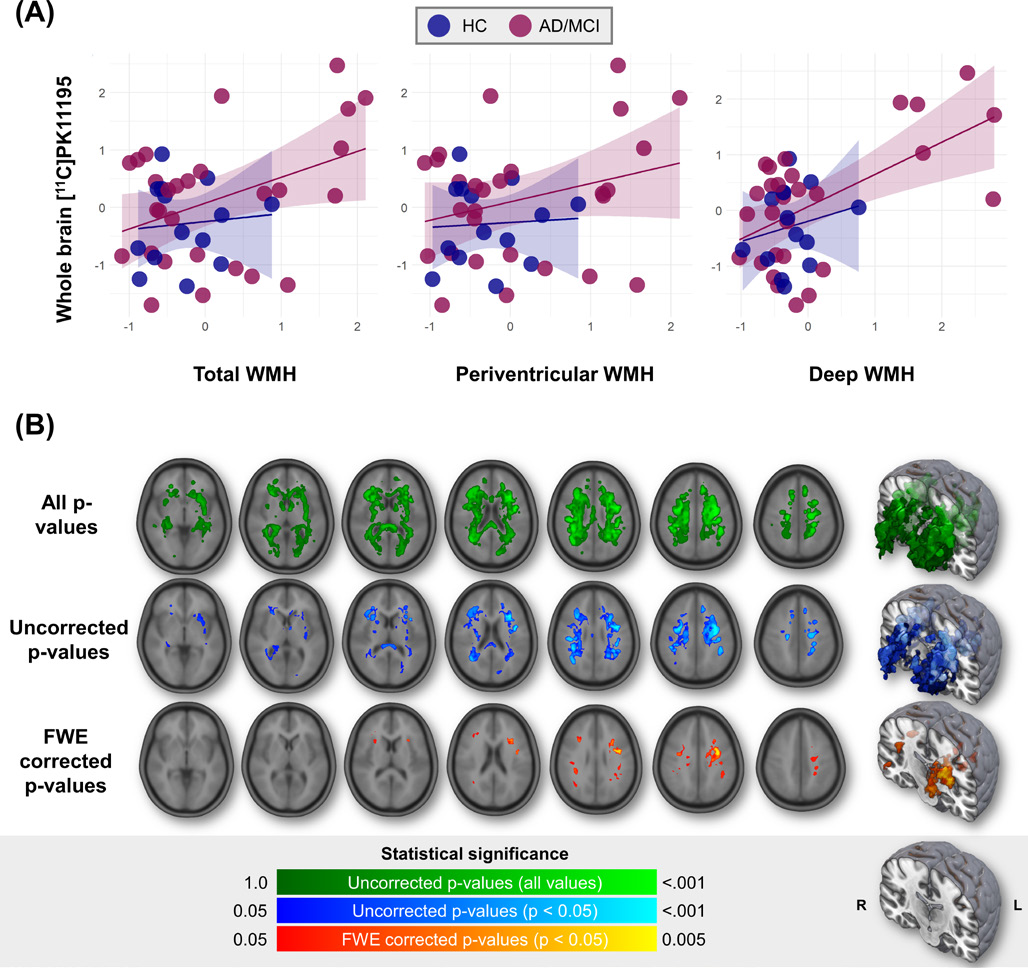
Association between whole brain [^11^C]PK11195 binding and WMH. (A) Scatterplot of whole brain [^11^C]PK11195 binding with WMH volumes. WMH values are residuals adjusted for sex, age, education, ACE-R score and scan interval. (B) Lesion probability maps of [^11^C]PK11195-related WMH spatial distribution, adjusted for sex, age, education, ACE-R score and scan interval. ACE-R, Addenbrooke’s Cognitive Examination-Revised; AD, Alzheimer’s disease; FWE, family-wise error; HC, healthy controls; MCI, mild cognitive impairment; WMH, white matter hyperintensities.

Whole-brain [^11^C]PK11195 binding was associated with total WMH volume in the overall sample after adjusting for age, sex and education and correcting for multiple comparisons (β=0.45, t=2.96, p=0.005). In both the overall sample and AD/MCI subgroup, whole-brain [^11^C]PK11195 binding was significantly associated with deep WMH (whole sample: β=0.59, t=4.91, p<0.001; AD/MCI: β=0.61, t=4.31, p<0.001), but not periventricular WMH (whole sample: β=0.33, t=2.01, p=0.052; AD/MCI: β=0.28, t=1.32, p=0.202) (figure 2A). Inclusion of hypertension or the composite vascular risk score into the regression model did not change these findings. Lesion probability maps were consistent with these findings, demonstrating that whole-brain [^11^C]PK11195 binding was associated with deep WMH, but not periventricular WMH (figure 2B).

**Figure 3:**
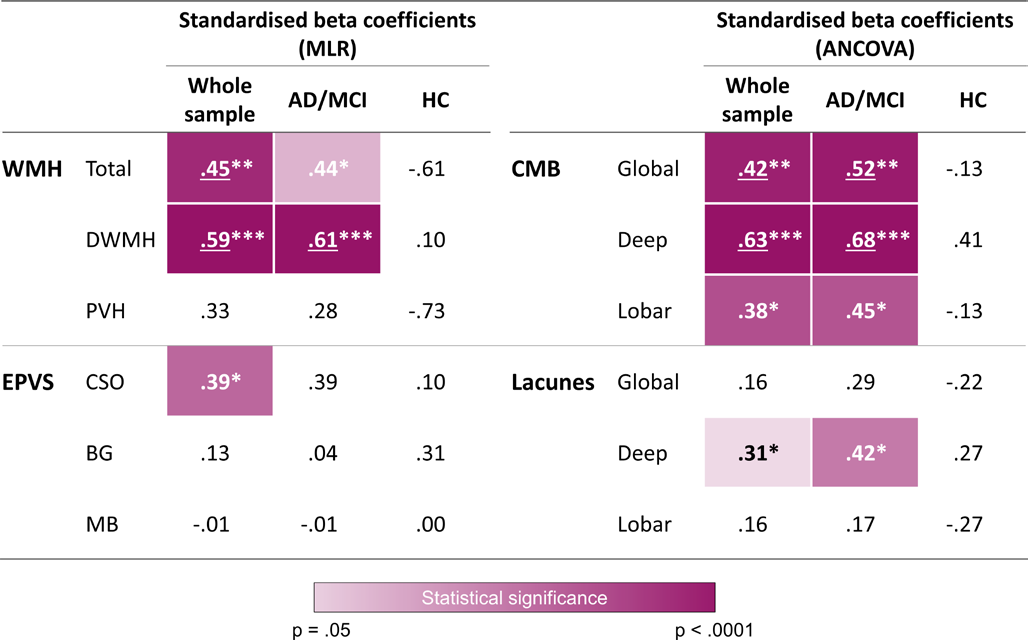
Graphical summary of associations between global [^11^C]PK11195 and SVD across imaging markers and regions. Values represent standardised beta coefficients of the individual markers, adjusted for sex, age, education, ACE-R and scan interval. Multiple linear regression was conducted for WMH and EPVS, while ANCOVA was conducted for presence/absence of CMB and lacunes. Colour scale represents p values, whereby darker shades signify smaller p values and unshaded (white) cells are not statistically significant. *p<0.05, **p<0.01, ***p<0.001; bold values represent statistical significance (p<0.05) before correcting for multiple comparisons; underlined values represent statistical significance (p<0.05) after false discovery rate correction for multiple comparisons (12 measures × 3 groups=36 comparisons). ACE-R, Addenbrooke’s Cognitive Examination-Revised; ANCOVA, analysis of covariance; AD/MCI, participants with Alzheimer’s disease or mild cognitive impairment; BG, basal ganglia; CMB, cerebral microbleeds; CSO, centrum semiovale; DWMH, deep white matter hyperintensities; HC, healthy controls; EPVS, enlarged perivascular spaces; MLR, multiple linear regression; PVH, periventricular white matter hyperintensities; MB, midbrain; SVD, small vessel disease; WMH, white matter hyperintensities.

The presence of CMB globally (whole sample: β=0.42, F(1,35)=10.09, p=0.003; AD/MCI: β=0.52, F(1,35)=12.16, p=0.002) and deep CMB (whole sample: β=0.63, F(1,35)=35.24, p<0.001; AD/MCI: β=0.68, F(1,35)=31.68, p<0.001) were associated with higher [^11^C]PK11195 binding in the whole sample and AD/MCI subgroup after correcting for multiple comparisons. Associations of [^11^C]PK11195 binding with lacunes and EPVS did not survive correction for multiple comparisons (figure 3).

### Association between whole brain [^11^C]PK11195 and global SVD burden scores

Global SVD burden was associated with higher [^11^C]PK11195 binding (β=0.46, t=3.31, p=0.002). In terms of SVD type, [^11^C]PK11195 was associated with both hypertensive arteriopathy (β=0.54, t=4.24, p<0.001) and CAA (β=0.46 t=3.25, p=0.003). These results were similar within the AD/MCI group, although only the hypertensive arteriopathy score was associated with [^11^C]PK11195 among healthy controls (β=0.60, t=3.02, p=0.019). Dominance analysis demonstrated that the hypertensive arteriopathy score (relative weight (RW)=0.19, dominance weight (DW)=0.17) significantly outperformed CAA (RW=0.03, DW <0.001) in predicting whole-brain [^11^C]PK11195.

### Association between global SVD burden scores and regional [^11^C]PK11195 distribution

**Figure 4:**
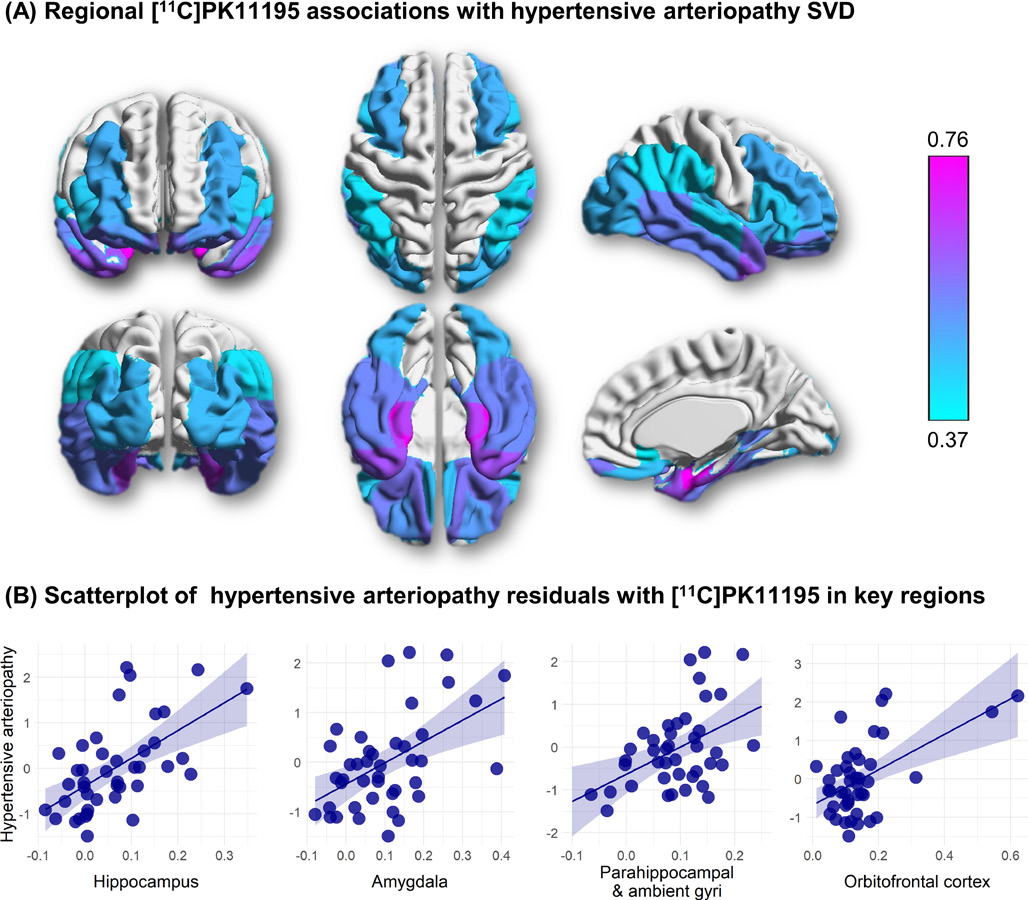
Associations between regional [^11^C]PK11195 binding and hypertensive arteriopathy (n=42). (A) β-weight brain mapping of associations between regional [^11^C]PK11195 binding and hypertensive arteriopathy. Coloured overlay of the Hammers atlas represents statistically significant elevation of [^11^C]PK11195 binding with hypertensive arteriopathy score controlling for sex, age, education, ACE-R score and scan interval and corrected for multiple comparisons. Colour gradient represents the strength of association (standardised β weights), increasing in magnitude from light blue to fuchsia. (B) Scatterplots of relationships between hypertensive arteriopathy (y-axis) and [^11^C]PK11195 binding in key regions of interest (x-axis). Hypertensive arteriopathy values are residuals, adjusted for sex, age, education, ACE-R and scan interval. ACE-R, Addenbrooke’s Cognitive Examination-Revised; SVD, small vessel disease.

ROI analysis indicated a significant relationship between hypertensive arteriopathy and [^11^C]PK11195 binding in 22 of the 37 Hammers atlas ROIs after FDR correction (figure 4). Associations with hypertensive arteriopathy score were especially robust with respect to [^11^C]PK11195 binding in the temporal lobe, particularly the medial temporal lobe (hippocampus: β=0.76, t=4.95, FDR-corrected p (p_FDR_) <0.001, amygdala: β=0.76, t=5.58, p_FDR_=0.002, parahippocampal and ambient gyri: β=0.66, t=3.90, p_FDR_ <0.001), anterior temporal lobe (β=0.59–0.63, t=3.53–3.93, p_FDR_=0.002–0.004) and the orbitofrontal cortex (β=0.51–0.57, t=3.53–4.30, p_FDR_=0.001–0.004).

CAA was related to higher [^11^C]PK11195 binding in 13 ROIs. The majority of these ROIs were a subset of hypertensive arteriopathy-related ROIs, including the temporal lobe (β=0.27–0.54, t=1.42–3.60, p_FDR_=0.017–0.205) and anteromedial orbitofrontal cortex (β=0.40–0.44, t=2.70–3.10, p_FDR_=0.027–0.033). CAA was related to some medial temporal lobe structures, but notably to a weaker extent (hippocampus: β=0.45, t=2.75, p_FDR_=0.032, amygdala: β=0.49, t=2.76, p_FDR_=0.032, parahippocampal and ambient gyri: β=0.27, t=1.42, p_FDR_=0.205(ns)) than in hypertensive arteriopathy. A structure in which [^11^C]PK11195 was uniquely associated with CAA, but not hypertensive arteriopathy, was the anterior cingulate (β=0.41, t=2.77, p_FDR_=0.032).

Dominance analysis was used to compare the relative importance of hypertensive arteriopathy and CAA scores as predictors of [^11^C]PK11195 binding in each ROI. The hypertensive arteriopathy score outperformed CAA in predicting [^11^C]PK11195 binding in 28 out of the 37 ROIs, while CAA did not dominate in any ROI. In line with the independent ROI analyses earlier, the greatest degree of hypertensive arteriopathy dominance over CAA was observed in the medial temporal lobe (hypertensive arteriopathy DW=0.25–0.26; CAA DW≤0.001–0.05) and orbitofrontal cortex (hypertensive arteriopathy DW=0.21–0.26; CAA DW=0.02–0.10). Regions in which neither SVD subtype dominated include the cingulate gyrus (anterior and posterior), subcallosal area and basal ganglia structures (nucleus accumbens, putamen and pallidum).

## Discussion

We found positive associations between in vivo neuroinflammation (microglia activation) and markers of SVD, particularly with the hypertensive subtype of SVD rather than CAA. Hypertensive arteriopathy outperformed CAA as a predictor of whole-brain [^11^C]PK11195 binding and across many ROI, especially the medial temporal lobe and orbitofrontal cortex.

The relationship between cerebral SVD and inflammation has been largely examined using peripheral blood markers of inflammation,^12^ which lack topological specificity to detect inflammation in the brain. As such, the present study expands on existing literature by examining topographical associations between neuroinflammation and different markers of SVD (WMH, EPVS, microbleeds, lacunes). Elevated neuroinflammation was associated with greater WMH and microbleed burden, while relationships with EPVS and lacunes were non-significant. Notably, the link between neuroinflammation and SVD was present only in AD/MCI patients, but not controls. This suggests that the interaction between SVD and neuroinflammation might have a key pathogenetic role in neurodegeneration rather than simply reflecting an age-related effect. Nevertheless, we acknowledge that our sample size was limited, and further studies are needed to confirm the interplay between SVD, neuroinflammation and neurodegeneration.

While the presence and directionality of causal mechanisms cannot be determined from this cross-sectional study, animal studies suggest that inflammation precedes, and may be causally related to, SVD.^12 31 32^ This is demonstrated especially by the reversal of WM damage observed following the use of drugs targeting inflammation and the endothelium, as well as the identification of a single-nucleotide polymorphism of a gene in SVD patients, a gene responsible for endothelial dysfunction when mutated in rats.^31 32^

**Figure 5:**
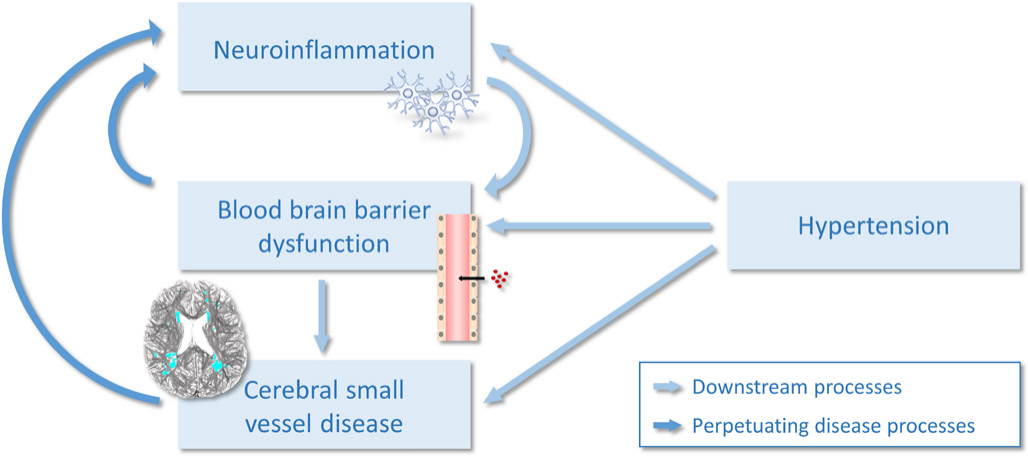
The vicious cycle of neuroinflammation and cerebral small vessel disease.

The relationship between [^11^C]PK11195 binding and SVD was more pronounced in hypertensive arteriopathy (rather than CAA). This differential association of neuroinflammation specifically with *hypertensive arteriopathy* implies that SVD subtypes involve distinct pathophysiological aetiologies and could shed light on the mechanisms linking inflammation and SVD. While CAA results from β-deposition in vessel walls, hypertensive SVD has stronger vascular involvements, implicating hypertension, blood pressure and leakages of the blood brain barrier (BBB).^14^ Hypertensive arteriopathy is characterised by lesions in deep subcortical regions like the basal ganglia. Anatomically, these areas are supplied by deep perforating arteries susceptible to twisting/looping and luminal narrowing, which can be exacerbated by hypertension. Inflammation is thought to promote vessel occlusion and endothelial dysfunction leading to increased BBB permeability—this in turn increases vessel wall fragility, which has been suggested to result in vessel ruptures and ‘leakage’ of red blood cells into the parenchyma, that is, microbleeds.^33^ In turn, the increased BBB permeability itself may also lead to a persistent immune response and a chronic neuroinflammatory state, resulting in a vicious cycle of pathological processes (figure 5). Taken altogether, neuroinflammation appears to be an early event in SVD. Although the precise sequence of events remains a topic of debate, evidence implicates neuroinflammation in the cascade of processes leading ultimately to the brain alterations seen in SVD.

Contrary to other SVD markers, neuroinflammation was related to EPVS in the centrum semiovale (CAA-related), rather than the basal ganglia (hypertension-related). This is perhaps unsurprising, given that EPVS itself has been considered a marker of neuroinflammation.^34^ Perivascular spaces are direct conduits for the drainage of interstitial fluids, making them vulnerable as a gateway for harmful foreign antigens to enter the brain. These foreign antigens, including inflammatory stimuli, lead to small vessel alterations and perivascular oedema. When these perivascular structures are compromised, manifested as MRI-visible EPVS, they are also proposed to be early markers of BBB dysfunction.^35^

Regional analysis showed that hypertensive arteriopathy was associated with widespread neuroinflammation, particularly in the medial temporal lobe (eg, hippocampus) and orbitofrontal cortex (including basal forebrain). CAA-related neuroinflammation was less widespread but overlapped with some regions of hypertensive arteriopathy-related inflammation such as the temporal lobe and basal forebrain. Notably, CAA was not related to hippocampal neuroinflammation which was robustly associated with hypertensive SVD. The exclusive involvement of hypertensive SVD in hippocampal inflammation may perhaps be attributed to the hippocampus’ (1) heightened sensitivity to vascular alterations resulting from its unique vascular architecture and perfusion characteristics^36^ and/or (2) significance as the earliest region to lose BBB integrity.^37^

Although CAA and hypertensive arteriopathy have been discussed as distinct SVD subtypes thus far, overlaps in pathology may exist, with suggestions that hypertensive arteriopathy may initiate and speed up the development of CAA and amyloid β accumulation.^38^

This study has implications on clinical decision-making and future research directions. In both clinical and research settings, SVD is commonly treated as a homogeneous construct. However, our present findings suggest different aetiological processes underlying two distinct forms of SVD, whereby neuroinflammation is implicated in hypertensive SVD more so than CAA. This provides a basis for clinicians and scientists to distinguish between the two SVD subtypes and better characterise their pathophysiological differences. Clinically, recognising the differential mechanisms behind the two forms of SVD would be particularly relevant for treatment decisions. One recent animal study demonstrated that microglial depletion prevented BBB leakage and hypertension-related cognitive impairment,^39^ although it is unclear whether the success of such interventions would extend to CAA-type SVD.

A strength of this study is the in vivo measurement of neuroinflammation using PET imaging of [^11^C]PK11195, which is an improvement over peripheral inflammatory markers given its ability to provide CNS neuroinflammatory measures and spatial information. Another key strength involves the comprehensive characterisation of SVD—all four major MRI markers of SVD were included, and region-specific burden was quantified for each SVD marker. In addition to global SVD burden,^27^ regional SVD measures allowed us to compute composite scores to examine different forms of SVD. Study limitations include its cross-sectional design, a relatively small sample size and inherent shortcomings of the [^11^C]PK11195 tracer such as low signal-to-noise TSPO binding. While further research should be conducted longitudinally on larger sample sizes using second-generation TSPO tracers such as [^11^C]PBR28, the use of [^11^C]PK11195 has its advantages, such as its selectivity for activated microglia over quiescent microglia and reactive astrocytes, its relative insensitivity to common TSPO polymorphisms compared with second-generation tracers and its well-established methods for kinetic analysis.^4 5 40^ To examine the directionality of their relationship, Mendelian randomisation could be used to investigate the effects of genetic variants associated with inflammation on SVD.

To the best of our knowledge, this study presents the first investigation on SVD and in vivo neuroinflammation (microglial activation) using [^11^C]PK11195 PET imaging. Our findings indicate associations between microglial activation and SVD, especially in hypertensive SVD. Further investigations (eg, longitudinal studies) are needed to establish the causal nature of the relationship observed, but our results suggest that targeting neuroinflammation might represent a potential novel therapeutic pathway in treating SVD.
