# Supplementary material for: In vivo neuroinflammation and cerebral small vessel disease in mild cognitive impairment and Alzheimer’s disease
Source: J Neurol Neurosurg Psychiatry. 2020 Sep 11;92(1):45–52. doi: 10.1136/jnnp-2020-323894 (PMC7803899; doi:10.1136/jnnp-2020-323894)
Supplement: Supplementary data [file jnnp-2020-323894supp001.pdf]

SUPPLEMENTARY MATERIALS: *In vivo* neuroinflammation and cerebral small vessel disease in mild cognitive impairment and Alzheimer's disease

Participant recruitment

MCI and AD participants were recruited from memory clinics in and around Cambridgeshire, via the Dementias and Neurodegeneration specialty of the UK Clinical Research Network (DeNDRoN) or the Join Dementia Research (JDR) platform (www.joindementiaresearch.nihr.ac.uk). Healthy controls were recruited from the JDR register, locally-held registers, and amongst friends, partners, and spouses of patients. Participants did not have any acute infectious or chronic symptomatic systemic inflammatory disorder (e.g., systemic lupus erythematosus, rheumatoid arthritis), or MRI contraindications.

All participants had mental capacity to take part in the study and provided written informed consent. The NIMROD protocol was approved by the National Research Ethic Service's East of England Cambridge Central Committee and the UK Administration of Radioactive Substances Advisory Committee.

MRI acquisition parameters

MRI scanning was conducted on Siemens 3T Tim Trio or Verio (Siemens Healthcare). Three-dimensional T1-weighted sequences (176 slices, 1.0mm thickness, repetition time (TR)=2300ms, echo time (TE)=2.98ms, field of view (FOV)=256x240mm², flip angle=9°, voxel size=1x1x1mm), T2 fluid-attenuated inversion recovery (FLAIR) images (75 slices, 2.0mm thickness, TR=12540ms, TE=132ms, flip angle=120°), and susceptibility-weighted imaging (SWI) (40 slices, 2.0mm thickness, TR=35ms, TE=20ms, flip angle=17°, acquisition matrix 256×240; voxel size=1×1×2mm) were obtained.

PET acquisition parameters

[^{11}C]PK11195, a ligand assessing microglial activation, was produced with high radiochemical purity (>95%), with specific activity of around 85 GBq/μmol at the end of synthesis. PET imaging was performed on a General Electric (GE) Advance PET scanner (GE Healthcare, Waukesha, WI) or a GE Discovery 690 PET/CT, with attenuation correction provided by a 15min 68Ge/68Ga transmission scan or a low dose CT scan, respectively. The emission protocol involved 75min of dynamic imaging (55 frames) starting concurrently with a 500 MBq [^{11}C]PK11195 injection. Each emission frame was reconstructed using the PROMIS 3-dimensional filtered back projection algorithm into a 128x128 matrix 30cm trans-axial FOV, with a trans-axial Hann filter cut-off at the Nyquist frequency.[1] Corrections were applied for randoms, dead time, normalization, scatter, attenuation, and sensitivity.

Semi-quantitative measures of small vessel disease

Periventricular and deep WMH were rated separately using the Fazekas scale: periventricular (0 = absent; 1 = "caps" or pencil-thin lining; 2 = smooth "halo"; 3 = irregular periventricular signal extending into the deep WM); deep (0 = absent; 1 = punctate foci; 2 = beginning confluence; 3 = large confluent areas).

All scans were rated by a single trained rater (A.L.), and 30% of all scans were rated by a second rater (J.D.S.), and 20% were re-rated by the main rater (A.L.) to assess intra-rater reliability. Both were blinded to clinical and diagnostic information. Inter-rater reliability (Cohen's kappa) were as follows: WMH – 0.80 (periventricular), 0.84 (deep); EPVS – 0.91 (centrum semiovale), 0.89 (basal ganglia); CMB – 0.81 (lobar), 0.79 (deep); lacunes – 0.70 (lobar), 0.74 (deep). Intra-rater reliability: WMH – 0.79 (periventricular), 0.87 (deep); EPVS – 0.90 (centrum semiovale), 0.87 (basal ganglia); CMB – 0.86 (lobar), 0.88 (deep); lacunes – 1.00 (lobar), 0.71 (deep). All discrepancies were discussed, and final ratings were arrived at by consensus.

Quantitative measure of WMH volume

WMH volumes were obtained using an automated script on the Statistical Parametric Mapping 8 (SPM8) suite (www.fil.ion.ucl.ac.uk/spm).[2] SPM8 was used to perform segmentation of T1-weighted images into grey matter (GM), WM, and cerebrospinal fluid (CSF), based on prior probability maps. Using the GM and WM maps, a brain mask was created and used to perform removal of non-brain matter from the FLAIR images. WMH maps were obtained using threshold-based segmentation at a threshold of 1.40 times the modal pixel intensity and segmented into periventricular and deep WMH. All generated segmentations were manually reviewed by experts (N.N., A.L., E.M.) and compared against raw FLAIR images to identify misclassifications. Misclassifications were manually corrected, and consensus was met amongst observers in cases of uncertainty.

Classification of SVD subtypes

For CAA, one point was awarded for each criterion met: (1) one or more lobar lacunes, (2) one or more lobar CMB, (3) EPVS score in centrum semiovale ≥ 2, (4) periventricular WMH Fazekas = 3 and/or deep WMH Fazekas ≥ 2. Similarly, for hypertensive arteriopathy: (1) one or more deep lacunes, (2) one or more deep CMB, (3) EPVS score in basal ganglia ≥ 2, (4) deep WMH Fazekas ≥ 2. While distinctive differences in the spatial distributions of lacunes, microbleeds, and EPVS between CAA and hypertensive arteriopathy have been well-documented,[3–8] differences in WMH patterns have not been well described in the literature. However, CAA has been associated to global WMH burden, which has been used in the computation CAA scores.[9] Etiologically, exclusive associations have been demonstrated between deep WMH (but not periventricular WMH) and vascular risk factors including hypertension, diabetes, obesity, and atrial fibrillation, suggesting a stronger vascular underpinning and overlap with hypertensive subtype of SVD [10]. These distinctions are supported by a recent genome-wide association study demonstrating distinct genetic

associations relating periventricular WMH with ischemic stroke, and deep WMH with vascular, astrocyte, and neuronal function.[11]

Statistical analysis

Normality of continuous data was tested using the Shapiro-Wilk Test. Parametric data (e.g., age, global [^{11}C]PK11195 binding, ACE-R, RAVLT) were analysed using either independent t-tests or analysis of variance (ANOVA), while nonparametric data (education, WMH volume, MMSE), were analysed using the Mann-Whitney U test. The Kruskal-Wallis test was used to analyse ordinal data such as Fazekas scores, EPVS scores, global SVD burden, CAA, and hypertensive arteriopathy scores. Chi-square tests of independence were used for group comparisons of categorical variables, i.e., the presence of CMB, lacunes, history of hypertension, hyperlipidaemia, diabetes mellitus, and smoking.

REFERENCES

1 Kinahan PE, Rogers JG. Analytic 3D image reconstruction using all detected events. *IEEE Trans Nucl Sci* 1989;**36**:964–8. doi:10.1109/23.34585

2 Firbank MJ, Minett T, O'Brien JT. Changes in DWI and MRS associated with white matter hyperintensities in elderly subjects. *Neurology* 2003;**61**:950–4. doi:10.1212/01.wnl.0000086375.33512.53

3 Greenberg SM, Vernooij MW, Cordonnier C, *et al.* Cerebral microbleeds: a guide to detection and interpretation. *Lancet Neurol* 2009;**8**:165–74. doi:10.1016/S1474-4422(09)70013-4

4 Wardlaw JM, Smith EE, Biessels GJ, *et al.* Neuroimaging standards for research into small vessel disease and its contribution to ageing and neurodegeneration. *Lancet Neurol* 2013;**12**:822–38. doi:10.1016/S1474-4422(13)70124-8

5 Pantoni L. Cerebral small vessel disease: from pathogenesis and clinical characteristics to therapeutic challenges. *Lancet Neurol* 2010;**9**:689–701. doi:10.1016/S1474-4422(10)70104-6

6 Pasi M, Boulouis G, Fotiadis P, *et al.* Distribution of lacunes in cerebral amyloid angiopathy and hypertensive small vessel disease. *Neurology* 2017;**88**:2162–8. doi:10.1212/WNL.0000000000004007

7 Charidimou A, Boulouis G, Haley K, *et al.* White matter hyperintensity patterns in cerebral amyloid angiopathy and hypertensive arteriopathy. *Neurology* 2016;**86**:505–11. doi:10.1212/WNL.0000000000002362

8 Charidimou A, Boulouis G, Pasi M, *et al.* MRI-visible perivascular spaces in cerebral amyloid angiopathy and hypertensive arteriopathy. *Neurology* 2017;**88**:1157–64. doi:10.1212/WNL.0000000000003746

9 Charidimou A, Martinez-Ramirez S, Reijmer YD, *et al.* Total magnetic resonance imaging burden of small vessel disease in cerebral amyloid angiopathy an imaging-pathologic study of concept validation. *JAMA Neurol* 2016;**73**:994–1001. doi:10.1001/jamaneurol.2016.0832

10 Moroni F, Ammirati E, Rocca MA, *et al.* Cardiovascular disease and brain health: Focus on white matter hyperintensities. IJC Hear. Vasc. 2018;**19**:63–9. doi:10.1016/j.ijcha.2018.04.006

11 Armstrong NJ, Mather KA, Sargurupremraj M, *et al.* Common Genetic Variation Indicates Separate Causes for Periventricular and Deep White Matter Hyperintensities. *Stroke* 2020;:2111–21. doi:10.1161/strokeaha.119.027544
